# Supplementary material for: Loss of bone morphogenetic protein-binding endothelial regulator causes insulin resistance
Source: Nat Commun. 2021 Mar 26;12:1927. doi: 10.1038/s41467-021-22130-2 (PMC7997910; doi:10.1038/s41467-021-22130-2)
Supplement: Supplementary file 1 — Supplementary Information [file 41467_2021_22130_MOESM1_ESM.pdf]

Supplementary Table 1. Mass spectrometry result shows representative proteins that potentially form complex with BMPER.

Gene ID	GeneSymbol	SRA	PSMs	iBAQ
3939	LDHA	S	118	0.38
4000	LMNA	S	100	0.17
8514	KCNAB2	S	49.2	0.12
4864	NPC1	S	19.2	0.05
54887	UHRF1BP1	S	13.5	0.02

S: high confidence

PSMs: total normalized peptide hits

iBAQ: total abundance of the gene

Supplementary Table 2. Metabolic parameters.

**Parameters**	**CC**		**HFD**		***db/db***	
	**GFP**	**BMPER**	**GFP**	**BMPER**	**GFP**	**BMPER**
**<u>Liver</u>**						
TG	8.44 ± 0.38	7.34 ± 0.91$^{(NS)}$	19.63 ± 0.26	13.98 ± 1.40$^{P=0.001}$	22.99 ± 1.90	18.88±0.63$^{P=0.04}$
DAG	2.98 ± 0.16	3.14 ± 0.16$^{(NS)}$	5.09 ± 0.27	3.11 ± 0.30$^{P=0.002}$	5.40 ± 0.29	3.28±0.33$^{P=0.001}$
BCAA	0.50 ± 0.05	0.51 ± 0.05$^{(NS)}$	0.64 ± 0.03	0.50 ± 0.09$^{(NS)}$	0.63 ± 0.06	0.52 ± 0.05$^{(NS)}$
Ceramide	0.16 ± 0.002	0.17 ± 0.002$^{(NS)}$	0.12 ± 0.001	0.12 ± 0.001$^{(NS)}$	0.12 ± 0.002	0.11 ± 0.002$^{(NS)}$
**<u>Skeletal muscle</u>**						
TG	4.88 ± 0.56	4.13 ± 0.63$^{(NS)}$	9.21 ± 0.59	10.08 ± 0.50$^{(NS)}$	10.33 ± 0.34	10.65 ± 0.12$^{(NS)}$
DAG	0.50 ± 0.07	0.54 ± 0.06$^{(NS)}$	0.88 ± 0.06	0.68 ± 0.08$^{(NS)}$	1.31 ± 0.07	0.71±0.11$^{P=0.002}$
BCAA	0.27 ± 0.03	0.24 ± 0.04$^{(NS)}$	0.41 ± 0.07	0.37 ± 0.04$^{(NS)}$	0.58 ± 0.08	0.48 ± 0.05$^{(NS)}$
Ceramide	0.08 ± 0.001	0.08 ± 0.001$^{(NS)}$	0.11 ± 0.002	0.11 ± 0.002$^{(NS)}$	0.11 ± 0.002	0.11 ± 0.002$^{(NS)}$

n=8 mice (**Liver**, CC or HFD; **Skeletal muscle**, CC or HFD), 5 mice (**liver**, *db/db*, GFP; **Skeletal muscle,** *db/db*, GFP), 7 mice (**liver**, *db/db*, BMPER; **Skeletal muscle,** *db/db*, BMPER). Data are presented as mean values ± SEM. NS, not significant. Units are mg/g tissue for TG and nmol/mg tissue for DAG, BCAA (Leucine, Isoleucine and Valine) and ceramide. Analysis was unpaired two-tailed Student's *t*-test.

Supplementary Table 3. Main reagent table.

REAGENT or RESOURCE	SOURCE	IDENTIFIER
**Antibodies**		
LRP1-CTD	Sigma	Cat#L2170
BMPER (Crossveinless-2/CV-2)	R&D Systems	Cat# AF2299
NPC1	Abcam	Cat# 124801
IRS-1	Cell signaling	Cat#2382
phospho- IRS1 (Tyr612)	Millipore	Cat#09-432
Akt	Cell signaling	Cat#9272
phospho-Akt (Ser473)	Cell signaling	Cat#9271
phospho-Akt (Thr308) (D25E6)	Cell signaling	Cat#13038
Insulin Receptor β (4B8)	Cell signaling	Cat#3025
β-Actin (C4) -HRP	Santa Cruz	Cat#sc-47778
Smad1	Cell signaling	Cat#9743
phospho- Smad1/Smad5/Smad8 (Ser463/465)	EMD Millipore	Cat#AB3848-I
Flag antibody	Sigma-Aldrich	Cat#2426
EZview Red ANTI-FLAG M2 Affinity Gel	Sigma-Aldrich	Cat#F2426
**Chemicals, Peptides, and Recombinant Proteins**		
60% high-fat diet (HFD)	Research Diets	Cat#D12492
Insulin	Novolin R	Cat#0169-1833-11
Mouse BMPER (Crossveinless-2) Protein	R&D Systems	Cat#2299-CV-050
Recombinant BMP2 protein	R&D Systems	Cat#355-BM/CF

Recombinant BMP2 protein	R&D Systems	Cat# 5020-BP-010
Advanced glycation endproduct-BSA	Sigma-Aldrich	Cat#121800-10MG-M
Palmitic acid	Sigma-Aldrich	Cat#P0500
Protein A/G Plus-agarose beads	Santa Cruz	Cat#sc-2003
iScript™ cDNA synthesis kit	Bio-Rad Laboratories	Cat#1708891
iTaq SYBR Green supermix	Bio-Rad Laboratories	Cat#1725121
TaqMan™ Universal PCR Master Mix	Thermo Scientific	Cat#4304437
Insulin solution	Santa Cruz	Cat# 11061-68-0
Cell maintenance supplements	Gibco	Cat# A15564
Tamoxifen	Sigma-Aldrich	Cat#T5648
Lipofectamine 2000	Thermo Scientific	Cat#11668019
Perfusion Medium	Thermo Scientific	Cat#17701038
Collagenase	Sigma-Aldrich	Cat#C5168
DMEM	Thermo Scientific	Cat#11965118
William's E Medium	Thermo Scientific	Cat#12551-032
Immobilon-P transfer membrane	Millipore	IPVH00010
Wash Medium	Thermo Scientific	Cat#17704-024
Percoll	GE Healthcare	Cat#17089101
OptiPrep density gradient medium	Sigma-Aldrich	Cat#1556
Penicillin & Streptomycin	Thermo Scientific	Cat#15140122
Fetal bovine serum (FBS)	Sigma-Aldrich	Cat#F0926
Horse serum	Thermo Scientific	Cat#16050114
Maintenance supplements	Thermo Scientific	Cat#CM4000
Chlorpromazine (CPM)	Sigma-Aldrich	Cat#C8138
Methyl-β-cyclodextrin (MCD)	Sigma-Aldrich	Cat#M7439

**Critical Commercial Assays**		
Insulin ELISA kit	Millipore	Cat#EZRMI-13K
RNA purification kit	QIAGEN	Cat#74104
Infinity Glucose kit	Thermo Scientific	Cat#TR15421
Albumin ELISA kit	Abcam	Cat#ab108792
Triglycerides reagent	Thermo Scientific	Cat#TR22421
DAG assay kit	Cell Biolabs	Cat#MET5028
BCAA assay kit	Cell Biolabs	Cat#MET5056
Ceramide ELISA kit	MyBiosource	Cat#MBS7255958
**Experimental Models: Cell Lines**		
Primary hepatocytes	This paper	N/A
HEK293 cells	ATCC	Cat#CRL-1573
Mouse embryonic fibroblasts	ATCC	Cat#SCRC-1008
Mouse heart endothelial cells	Cell Biologics	Cat#C57-6024
Mouse lung endothelial cells	Cell Biologics	Cat#C57-6011
Mouse liver endothelial cells	Cell Biologics	Cat#C57-6017
**Experimental Models: Organisms/Strains**		
Mouse: B6.Cg-Tg (CAG-cre/ Esr1*) 5Amc/J (CAG-CreER$^{+/-}$)	Jackson Laboratories	JAX: 004682
Mouse: Cdh5(PAC)-CreERT2 (Cdh5-CreER$^{+/-}$)	Dr. Ralf H. Adams from Max Planck Institute for Molecular Biomedicine, Germany	N/A
Mouse: C57BL/6	Jackson Laboratories	JAX: 000664
Mouse: IR^{flox}	Jackson Laboratories	JAX: 006955
Mouse: B6 db	Jackson Laboratories	JAX: 000697
**Recombinant DNA**		
Npc1 mouse shRNA plasmid	Origene	Cat#TL501501

Npc2 mouse shRNA plasmid	Origene	Cat#TL504048
pCMV-Tag2 vector	Agilent	Cat#211172
Scrambled shRNA plasmid	OriGene	Cat#TR30021
pLenti-C-Flag-DDK-BMPER	This paper	N/A
Ad-CMV-iCre	Vector Biolabs	Cat#1045

Supplementary Table 4. Sequence information for qPCR primers.

Gene	Forward primer	Reverse primer
G6Pase	5'- tctgtcccggatctaccttg-3'	5'- gaaagtttcagccacagcaa-3'
PEPCK	5'- ggagtacccattgagggtatcat-3'	5'- gctgagggcttcatagacaag-3'
GK	5'- cagatgctggatgacagagc-3'	5'- gccaggatctgctctaccttt-3'
SREBP1	5'- acaagattgtggagctcaaagac-3'	5'- tgcgcaagacagcagattta-3'
IL1β	5'- agttgacggaccccaaaag-3'	5'- agctggatgctctcatcagg-3'
IL6	5'- gctaccaaactggatataatcagga-3'	5'- ccaggtagctatggtactccagaa-3'
TNFα	5'- ctgtagcccacgtcgtagc-3'	5'- ttgagatccatgccgttg-3'
β-Actin	5'- ccaaccgtgaaaagatgacc-3'	5'- accagaggcatacagggaca-3'
BMPER	5'- ggctgagccatgtgtcct-3'	5'- cgcacctcagactctgtcac-3'
BMP2	5'- agatctgtaccgcaggcact-3'	5'- gttcctccacggcttcttc-3'
BMP4	5'- gatctttaccggctccagtct-3'	5'- tgggatgttctccagatgttc-3'
BMP6	5'- actgactagcgcgcagga-3'	5'- tgtggggagaactccttgtc-3'
BMP7	5'- cgagaccttccagatcacagt-3'	5'- cagcaagaagaggtccgact-3'
BMP9	5'- ggaagctgtgggtagatgacc-3'	5'- caagtcggtggggatgat-3'
BMPR2	5'- gagccctcccttgacctg-3'	5'- gtatcgaccccgtccaatc-3'

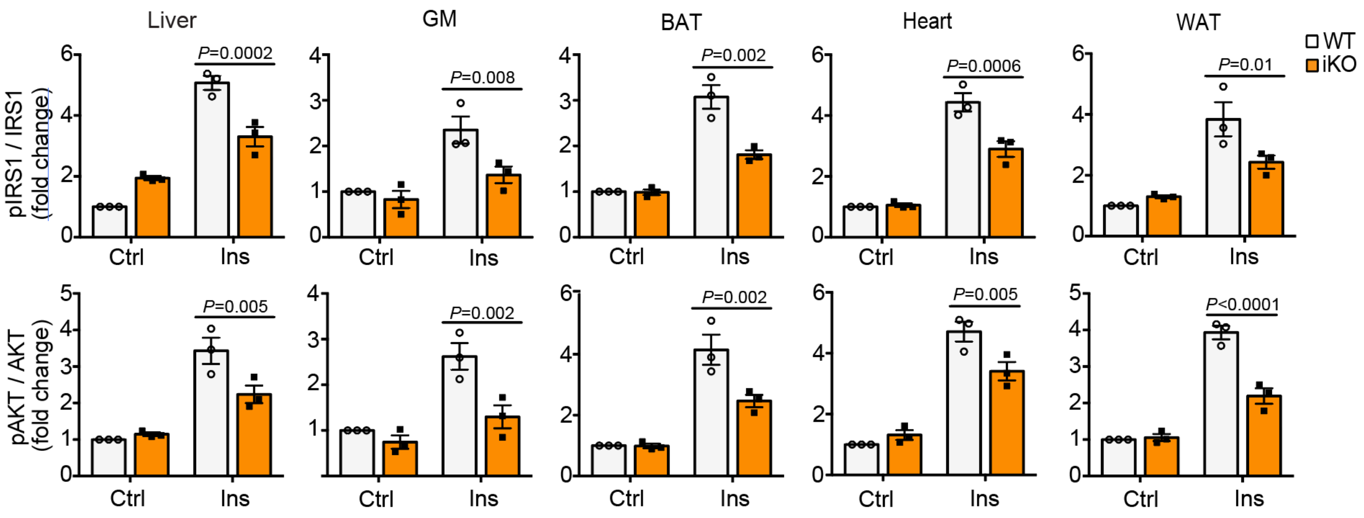

Supplementary Figure 1. Quantification for Figure 1l. n=3 mice. Data are presented as mean values ± SEM. Analysis was two-way ANOVA followed by Fisher's LSD multiple comparison test.

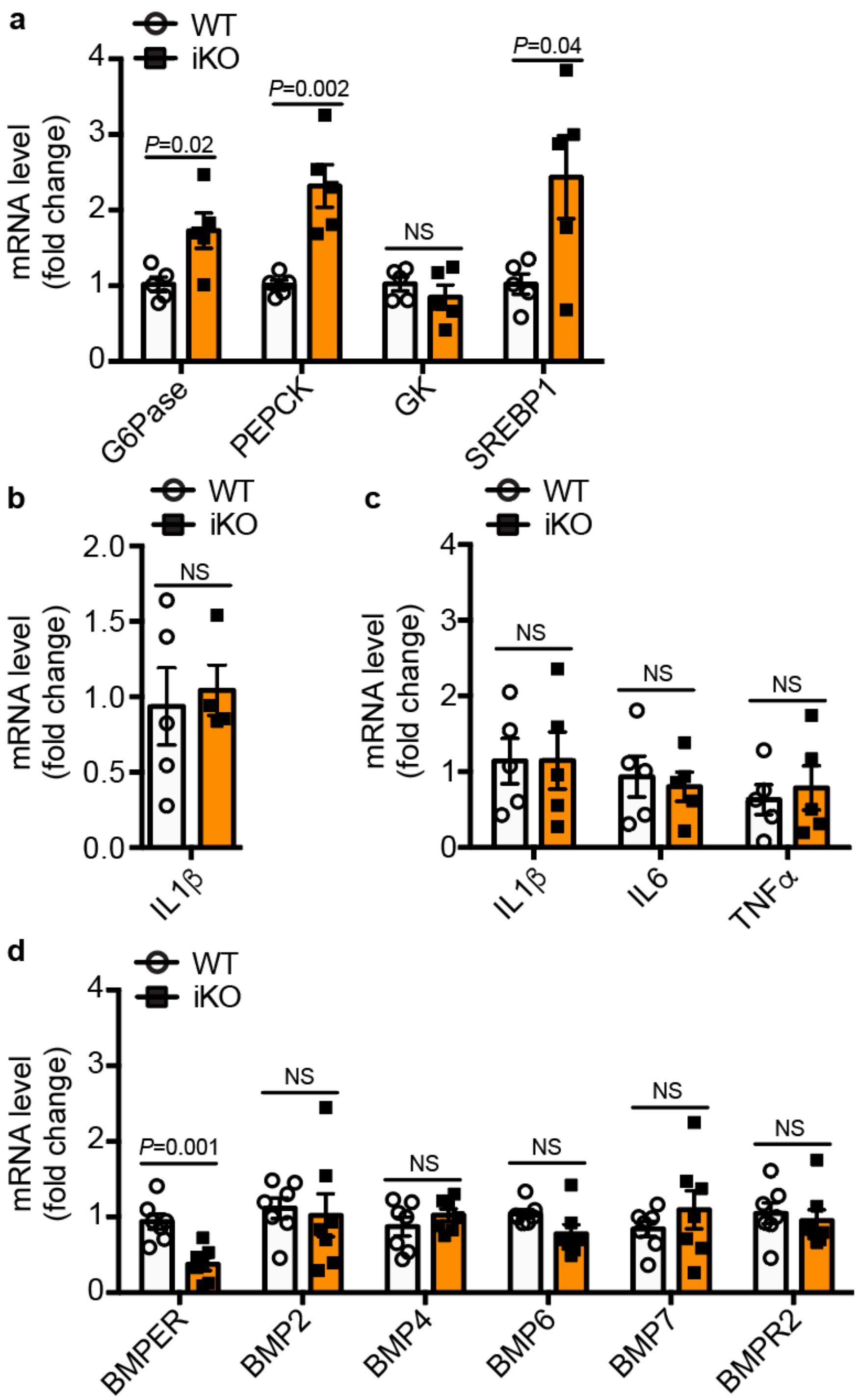

Supplementary Figure 2. Gene expression changes in liver and WAT tissues of BMPER iKO mice. (**a-d**) Real-time PCR assays were performed for indicated genes in the liver (**a, b, d**) and WAT (**c**) of BMPER iKO and their littermate control (WT) mice. n=5 (**a, c**), **5** (**b**, WT), 4 (**b**, iKO) and 7 (**d**). Data are presented as mean values ± SEM. NS, not significant. Analysis was unpaired two-tailed Student's *t*-test.

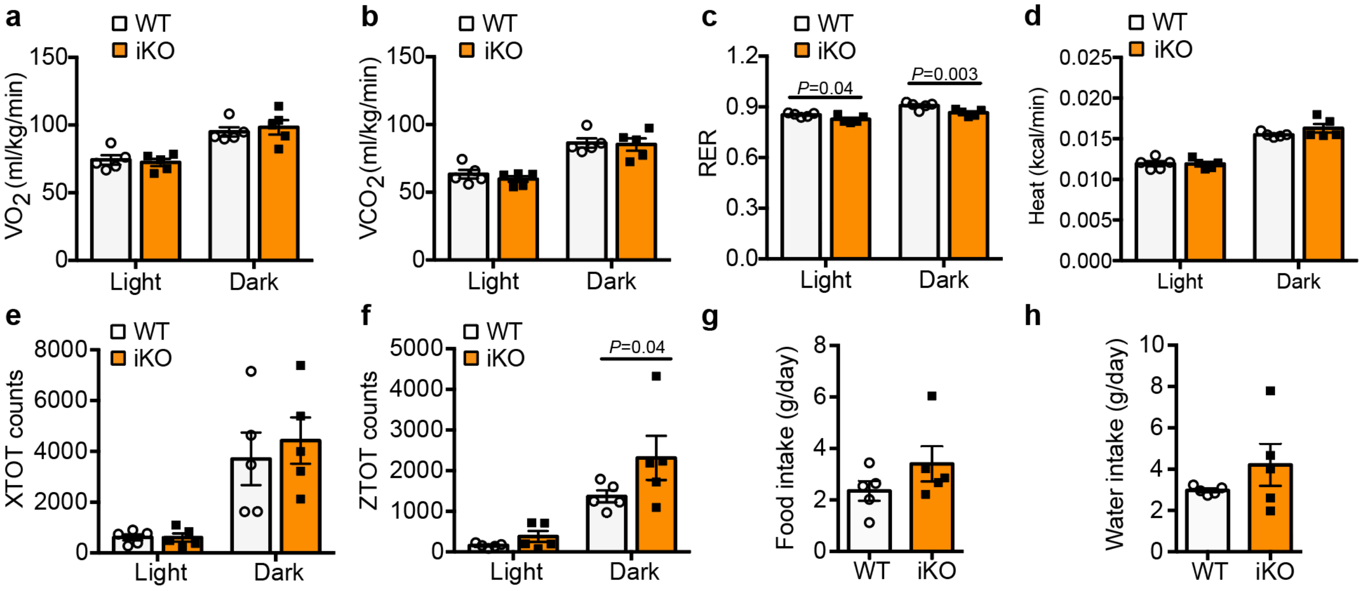

Supplementary Figure 3. CLAMS studies with BMPER iKO mice. Indirect calorimetry studies were performed to evaluate VO_2 (**a**), VCO_2 (**b**), respiratory exchange ratio (RER, **c**), heat (**d**), locomotor activity in x axis (XTOT, **e**) and z axis (ZTOT, **f**), daily food (**g**) and water intake (**h**). n=5 mice (**a-h**). Data are presented as mean values ± SEM. Analysis was two-way ANOVA followed by Fisher's LSD multiple comparison test (for **a-f**) and unpaired two-tailed Student's *t*-test (for **g, h**).

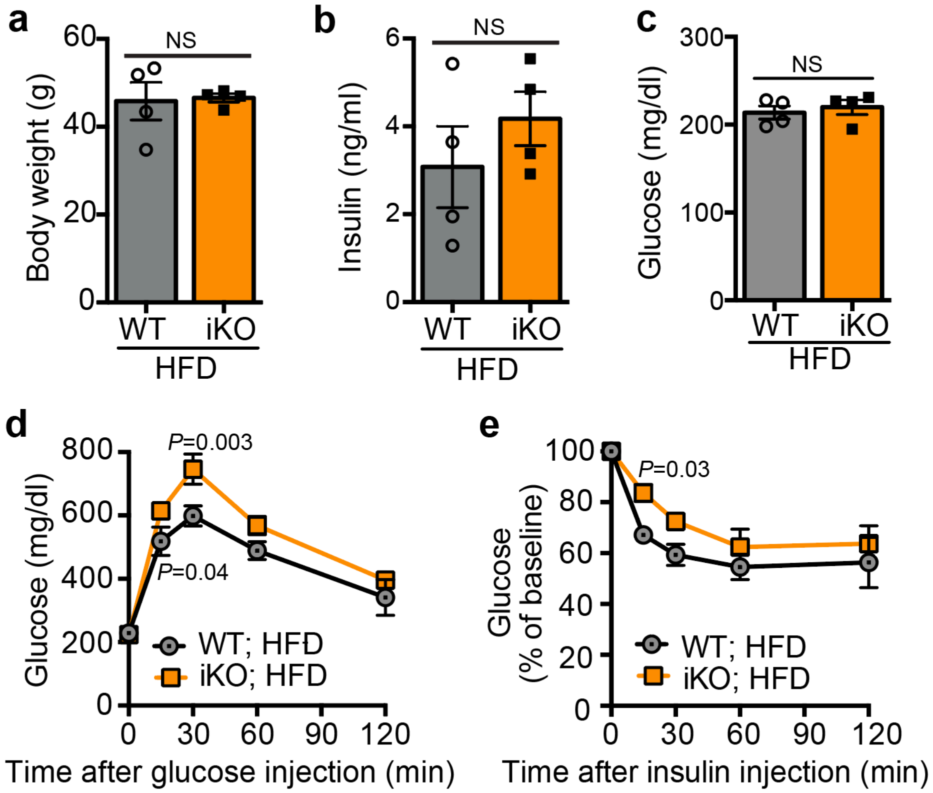

Supplementary Figure 4. BMPER depletion exacerbates glucose responses in HFD-fed mice. Metabolic studies were performed with BMPER iKO and WT mice following eight weeks of HFD feeding. (**a**) Body weight. (**b-c**) Fasted insulin and glucose. (**d-e**) Glucose and insulin tolerance tests. HFD, high-fat diet. n= 4 mice (**a-e**). Data are presented as mean values ± SEM. NS, not significant. Analysis was unpaired two-tailed Student's *t*-test (for **a-c**) and two-way ANOVA followed by Fisher's LSD multiple comparison test (for **d, e**).

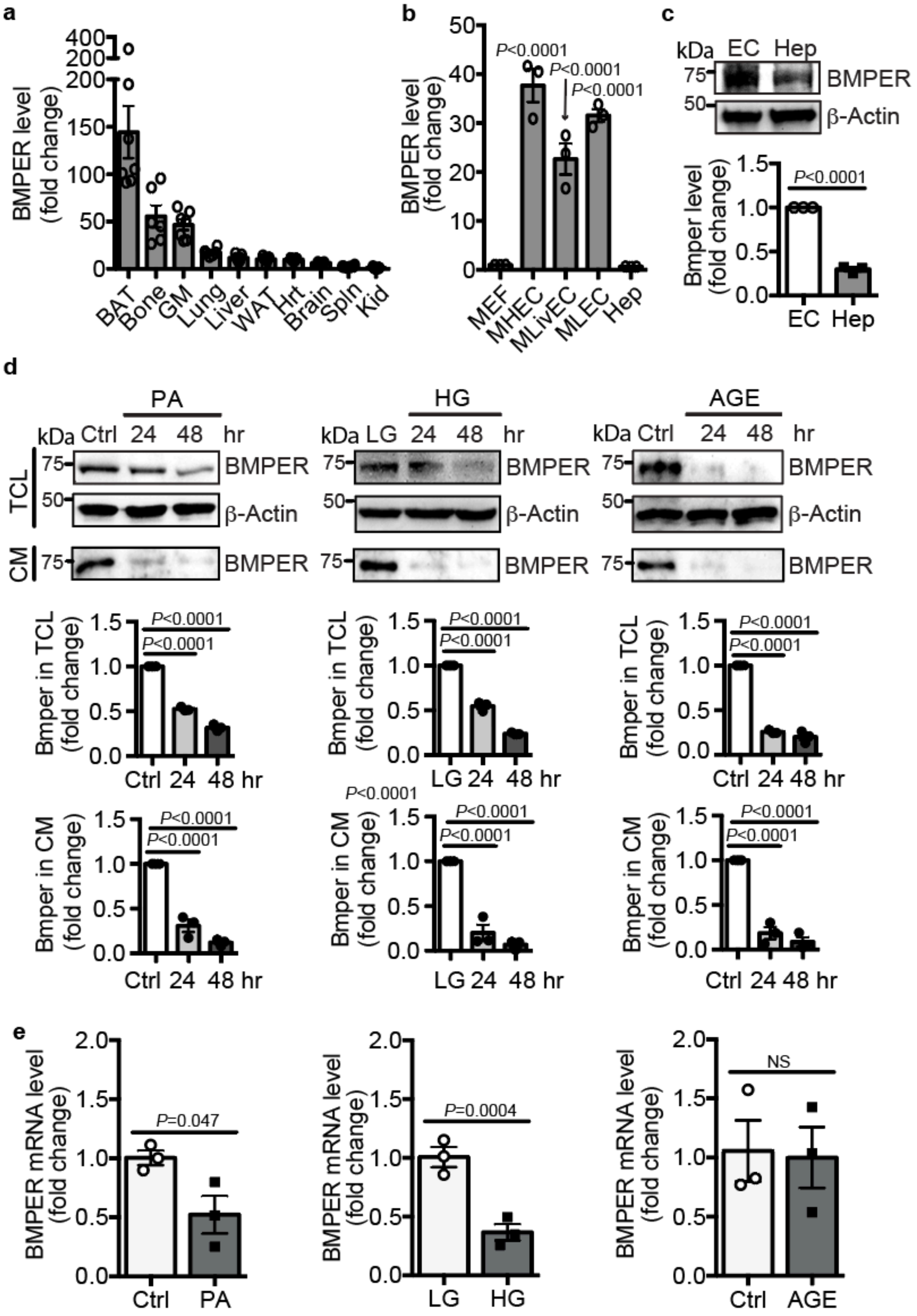

Supplementary Figure 5. BMPER expression in ECs is downregulated by metabolic stress. (**a**) BMPER mRNA levels in different tissues. BAT, brown adipose tissue. GM, gastrocnemius muscle. WAT, white adipose tissue. Hrt, heart. Spln, spleen. Kid, kidney. (**b**) BMPER mRNA levels in different primary cells. MEF, mouse embryonic fibroblast. MHEC, mouse heart EC. MLivEC, mouse liver EC. MLEC,

mouse lung EC. Hep, hepatocytes. (**c**) BMPER protein levels in EC and hepatocytes. Cell lysates of MLECs and hepatocytes were subjected for Western blotting assays. (**d-e**) BMPER protein level in EC total cell lysates (TCL) and conditioned medium (CM, **d**) and its mRNA (**e**) levels in ECs were decreased. MLivECs were treated with palmitic acids (PAs, 0.2 mM), high glucose (HG, 25 mM) or advanced glycation end products (AGEs, 100 ug/ml). LG, low glucose (5 mM supplemented with 20 mM mannitol as the osmotic control). BSA was used as the control (Ctrl) for PA and AGE treatments. TCL and CM were subjected for Western blotting or real time PCR assays. n=6 mice (**a,** bone, lung, spleen), 7 mice (**a**, other tissues), 3 experiments (**b-e**). Data are presented as mean values ± SEM. NS, not significant. Analysis was one-way ANOVA followed by Fisher's LSD multiple comparison test (for **b, d**) or unpaired two-tailed Student's *t*-test (for **c, e**).

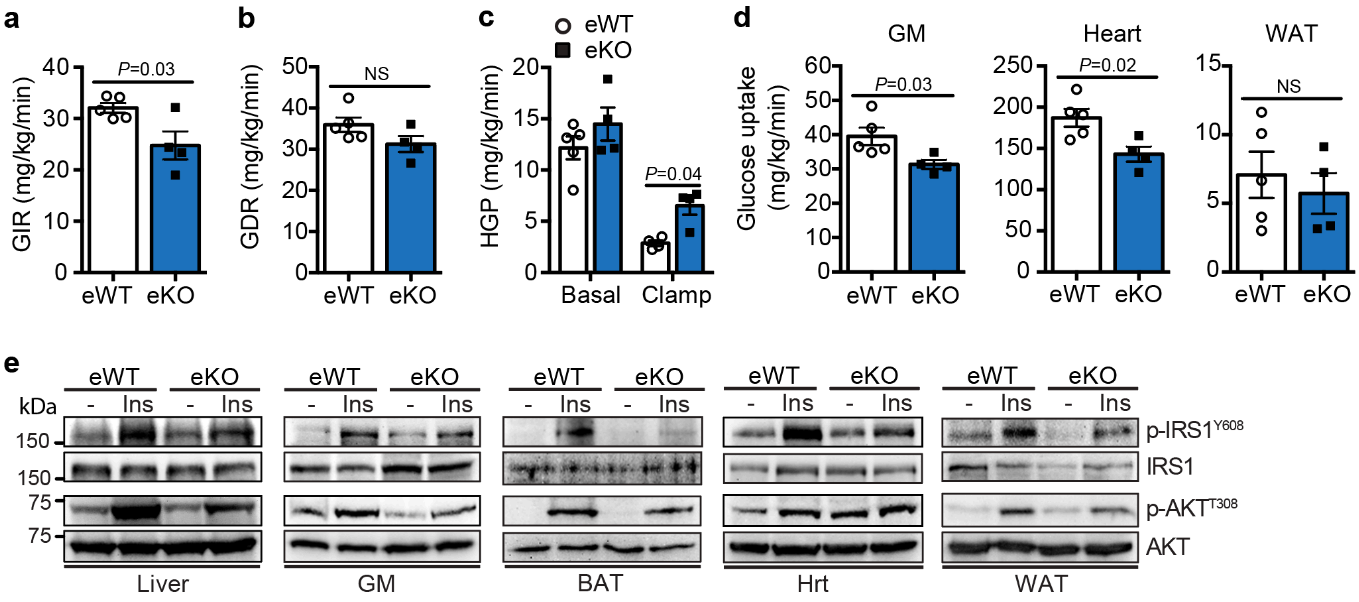

Supplementary Figure 6. BMPER depletion in ECs leads to insulin resistance. Clamp studies were performed with BMPER eKO and eWT mice at four months after tamoxifen injection. (**a**) Glucose infusion rate (GIR). (**b**) Glucose disposal rate (GDR). (**c-d**) Hepatic glucose production (HGP; **c**) and glucose uptake in peripheral tissues (**d**) were analyzed with hyperinsulinemic-euglycemic clamps. (**e**) Insulin signaling was blunted in BMPER eKO mice. Insulin (Ins, 0.5 hour) was injected (*i.p.*) into BMPER eKO and eWT mice. Indicated tissues were used for Western blotting. GM, gastrocnemius muscle; WAT, white adipose tissue. BAT, brown adipose tissue. Hrt, heart. n=5 mice (**a-d**, eWT), 4 mice (**a-d**, eKO). Data are presented as mean values ± SEM. NS, not significant. Analysis was unpaired two-tailed Student's *t*-test (for **a, b, d**) and two-way ANOVA followed by Fisher's LSD multiple comparison test (for **c**).

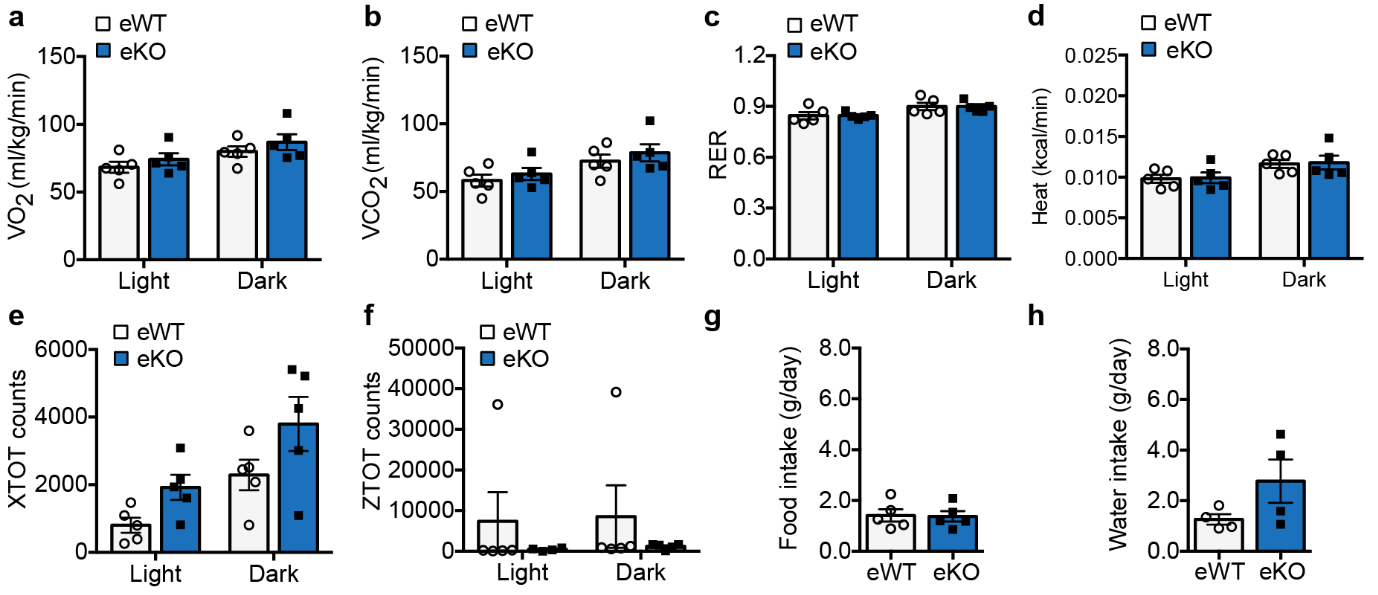

Supplementary Figure 7. CLAMS studies with BMPER eKO mice. Indirect calorimetry studies were performed to evaluate VO_2 (**a**), VCO_2 (**b**), respiratory exchange ratio (RER, **c**), heat (**d**), locomotor activity in x axis (XTOT, **e**) and z axis (ZTOT, **f**), daily food (**g**) and water intake (**h**). n=5 mice (**a-h**). Data are presented as mean values ± SEM. Analysis was two-way ANOVA followed by Fisher's LSD multiple comparison test (for **a-f**) and unpaired two-tailed Student's *t*-test (for **g, h**).

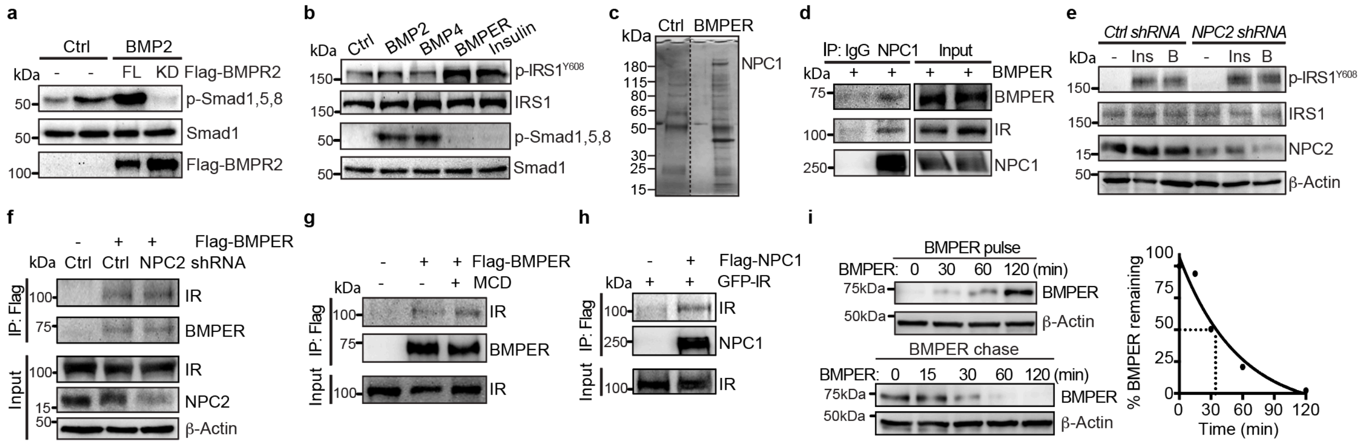

Supplementary Figure 8. The regulation of BMPER-promoted insulin signaling pathway. (**a**) Hepatocytes were transduced with adenovirus of flag-tagged BMPR2 full length (FL) or kinase-dead mutant (KD). Cells were then treated with BMP2 (4 nM, 1 hr). (**b**) Hepatocytes were treated with BMP2 (1 hr), BMP4 (1 hr), BMPER (1 hr) and insulin (30 min) and lysates were subjected for Western blotting assays. (**c**) NPC1 was identified in BMPER-bound complex in HEK293 cells. Cells were treated with BMPER (30 nM) and lysates were immunoprecipitated (IPed) with anti-BMPER antibody and stained with Coomassie blue. Selected bands were subjected for mass spectrometry analysis. (**d**) BMPER was identified in BMPER-bound complex in hepatocytes. Cells were treated with BMPER and lysates were IPed with anti-NPC1 antibody or IgG as the control. (**e**) Hepatocytes were transduced with *NPC2* shRNA lentivirus and then treated with BMPER (B, 1 hr) or insulin (Ins, 30 min). Cell lysates were subjected for Western blotting assays. (**f-g**) Hepatocytes were transduced with *NPC2* shRNA lentivirus (**f**) or pretreated with methyl-β-cyclodextrin (MCD, 10 mM, 30 min, **g**) and then treated with flag-tagged BMPER. Cell lysates were subjected for IP for BMPER-interacting proteins. (**h**) Co-IP of Flag-tagged NPC1 and GFP-tagged IR in HEK293 cells. (**i**) Hepatocytes were pulsed with Flag-BMPER (100 nM, 7.2 ng) for indicated time periods (left top panel), and then chased at 4°C (left bottom panel). Right panel is graphic representation of normalized intracellular BMPER level from the cold chase. n=3 experiments (**i**).

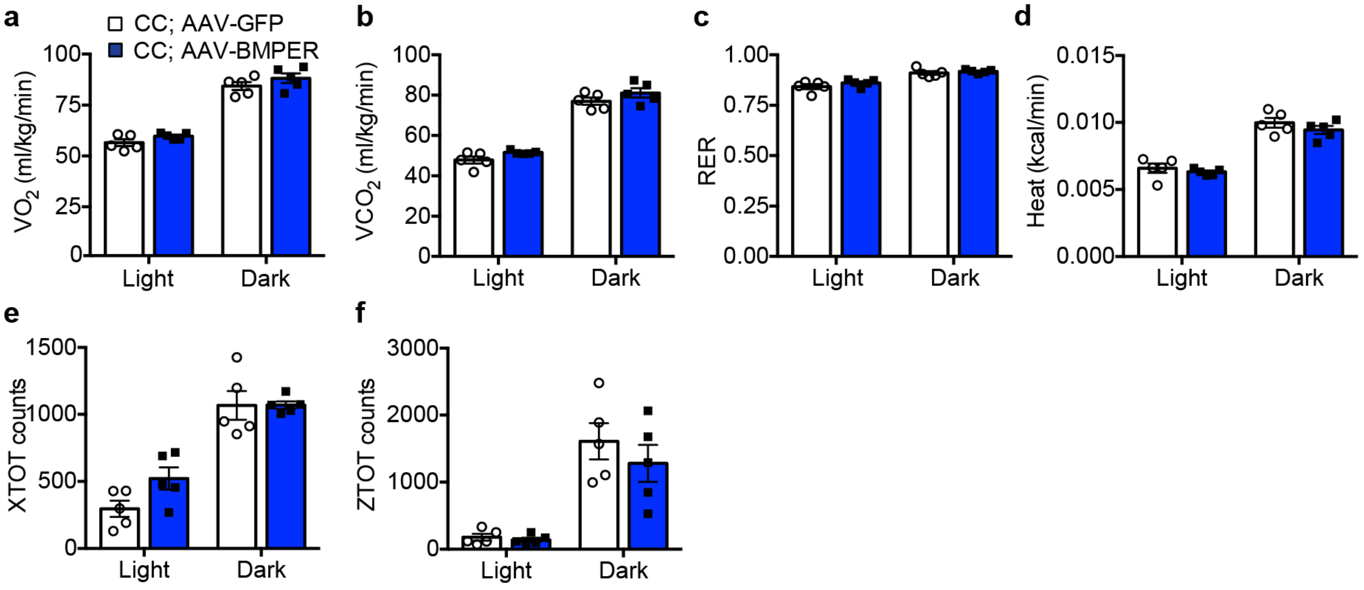

Supplementary Figure 9. CLAMS studies with AAV-BMPER injected mice following control chow-feeding. Indirect calorimetry studies were performed to evaluate VO_2 (**a**), VCO_2 (**b**), respiratory exchange ratio (RER, **c**), heat (**d**), locomotor activity in x axis (XTOT, **e**) and z axis (ZTOT, **f**). n=5 mice (**a-f**). Data are presented as mean values ± SEM. Analysis was two-way ANOVA followed by Fisher's LSD multiple comparison test.

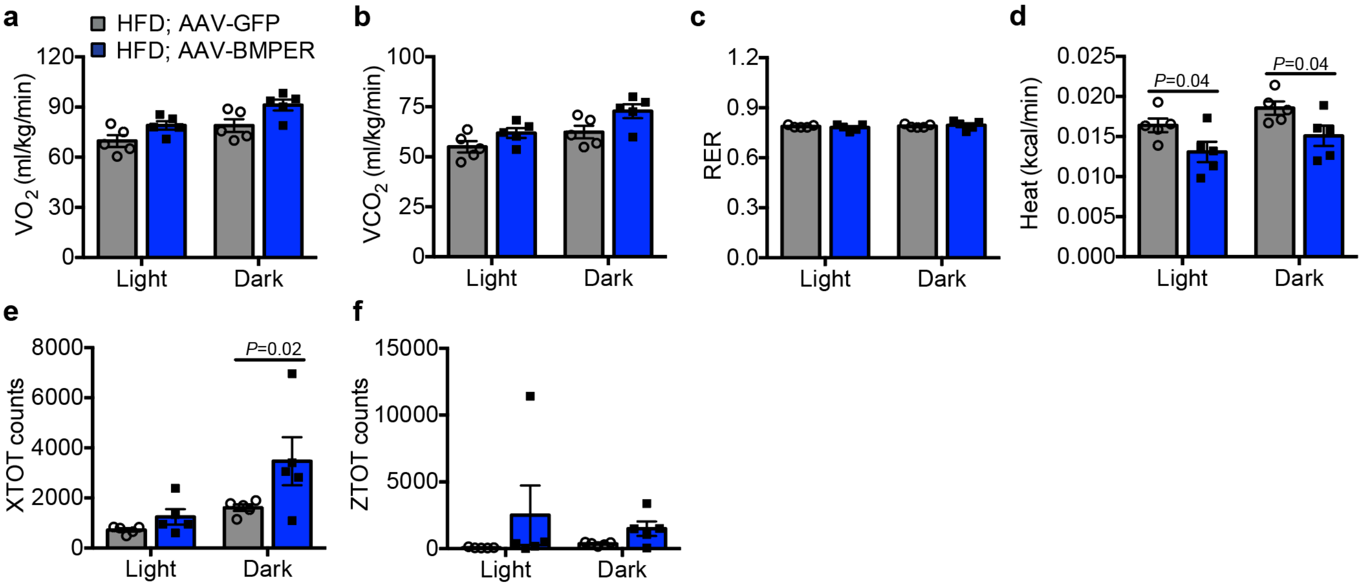

Supplementary Figure 10. CLAMS studies with AAV-BMPER injected mice following HFD feeding. Indirect calorimetry studies were performed to evaluate VO_2 (**a**), VCO_2 (**b**), respiratory exchange ratio (RER, **c**), heat (**d**), locomotor activity in x axis (XTOT, **e**) and z axis (ZTOT, **f**). n=5 mice (**a-f**). Data are presented as mean values ± SEM. Analysis was two-way ANOVA followed by Fisher's LSD multiple comparison test.

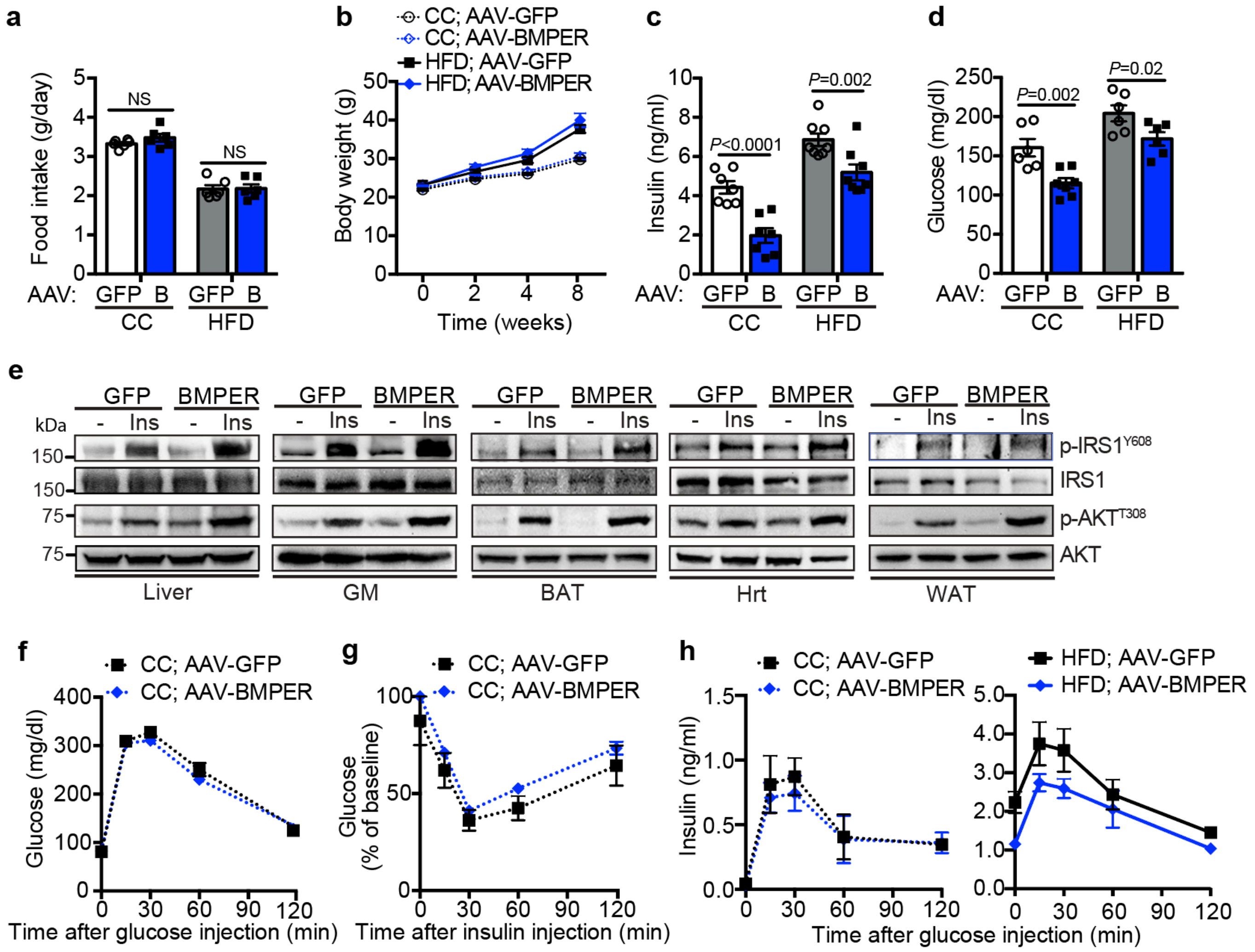

Supplementary Figure 11. AAV-BMPER improves glucose responses in DIO mice. AAV-BMPER or AAV-GFP was injected into C57BL/6 mice and then fed them HFD for eight weeks. (**a**) Food intake in AAV-GFP or AAV-BMPER (B) injected mice that were fed HFD or CC diet. (**b**) Body weight. (**c-d**) Fed insulin and glucose. (**e**) Insulin signaling was enhanced in AAV-BMPER-injected mice. Insulin (Ins, 0.5 hour) was injected (*i.p.*) into AAV-BMPER and GFP-injected mice. Indicated tissues were used for Western blotting. (**f-g**) Glucose and insulin tolerance tests. (**h**) Insulin levels during glucose tolerance tests. n=6 mice (**a**), 7-8 mice (**b**), 7 mice (**c,** CC), 8 mice (**c**, HFD), 6-7 mice (**d**, CC), 6 mice (**d**, HFD), 7 mice (**f-g**, AAV-GFP), 8 mice (**f-g,** AAV-BMPER) and 5 mice (**h**). Data are presented as mean values ± SEM. NS, not significant. Analysis was two-way ANOVA followed by Fisher's LSD multiple comparison test.

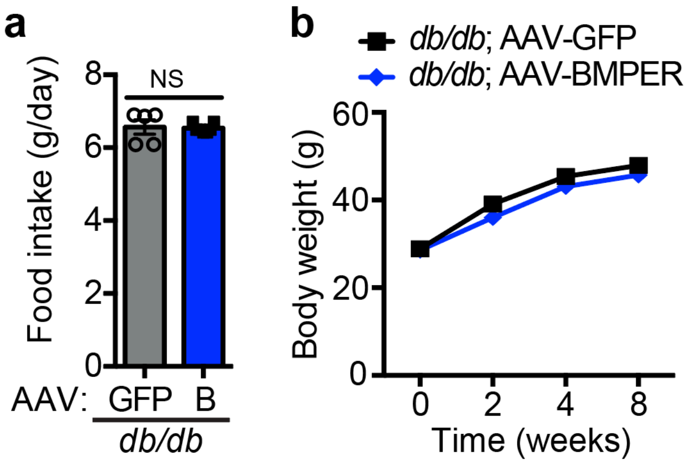

Supplementary Figure 12. AAV-BMPER improves glucose responses in *db/db* mice. AAV-BMPER or AAV-GFP was injected into 5-week-old *db/db* mice and then monitored for eight weeks. (**a**) Food intake in AAV-GFP or AAV-BMPER (B)- injected *db/db* mice. (**b**) Body weight. (**c-d**) Fed insulin and glucose. n=5 mice (**a**, **b, c**, AAV-GFP), 7 mice (**a**, **b**, AAV-BMPER), 6 mice (**c**, AAV-BMPER) and 5 mice (**d**). Data are presented as mean values ± SEM. NS, not significant. Analysis was unpaired two-tailed Student's *t*-test (for **a, c, d**) or two-way ANOVA followed by Fisher's LSD multiple comparison test (for **b**).
