# Supplementary material for: Loss of bone morphogenetic protein-binding endothelial regulator causes insulin resistance
Source: Nat Commun. 2021 Mar 26;12:1927. doi: 10.1038/s41467-021-22130-2 (PMC7997910; doi:10.1038/s41467-021-22130-2)
Supplement: Supplementary file 3 — Reporting Summary [file 41467_2021_22130_MOESM3_ESM.pdf]

nature research

Corresponding author(s): Xinchun Pi

Last updated by author(s): Jan. 20, 2021

Reporting Summary

Nature Research wishes to improve the reproducibility of the work that we publish. This form provides structure for consistency and transparency in reporting. For further information on Nature Research policies, see Authors & Referees and the Editorial Policy Checklist.

Statistics

For all statistical analyses, confirm that the following items are present in the figure legend, table legend, main text, or Methods section.

n/a	Confirmed	
☐	☒	The exact sample size (*n*) for each experimental group/condition, given as a discrete number and unit of measurement
☐	☒	A statement on whether measurements were taken from distinct samples or whether the same sample was measured repeatedly
☐	☒	The statistical test(s) used AND whether they are one- or two-sided<br>*Only common tests should be described solely by name; describe more complex techniques in the Methods section.*
☐	☒	A description of all covariates tested
☐	☒	A description of any assumptions or corrections, such as tests of normality and adjustment for multiple comparisons
☐	☒	A full description of the statistical parameters including central tendency (e.g. means) or other basic estimates (e.g. regression coefficient) AND variation (e.g. standard deviation) or associated estimates of uncertainty (e.g. confidence intervals)
☐	☒	For null hypothesis testing, the test statistic (e.g. *F*, *t*, *r*) with confidence intervals, effect sizes, degrees of freedom and *P* value noted<br>*Give P values as exact values whenever suitable.*
☒	☐	For Bayesian analysis, information on the choice of priors and Markov chain Monte Carlo settings
☒	☐	For hierarchical and complex designs, identification of the appropriate level for tests and full reporting of outcomes
☐	☒	Estimates of effect sizes (e.g. Cohen's *d*, Pearson's *r*), indicating how they were calculated

Our web collection on statistics for biologists contains articles on many of the points above.

Software and code

Policy information about availability of computer code

Data collection	n/a
Data analysis	n/a

For manuscripts utilizing custom algorithms or software that are central to the research but not yet described in published literature, software must be made available to editors/reviewers. We strongly encourage code deposition in a community repository (e.g. GitHub). See the Nature Research guidelines for submitting code & software for further information.

Data

Policy information about availability of data

All manuscripts must include a data availability statement. This statement should provide the following information, where applicable:

- Accession codes, unique identifiers, or web links for publicly available datasets
- A list of figures that have associated raw data
- A description of any restrictions on data availability

All main and supplementary raw data are available.

Field-specific reporting

Please select the one below that is the best fit for your research. If you are not sure, read the appropriate sections before making your selection.

☒ Life sciences ☐ Behavioural & social sciences ☐ Ecological, evolutionary & environmental sciences

For a reference copy of the document with all sections, see nature.com/documents/nr-reporting-summary-flat.pdf

Life sciences study design

All studies must disclose on these points even when the disclosure is negative.

Sample size	No statistical methods were used to predetermine the sample size.
Data exclusions	Using GraphPad Prism software, individual technical replicates were excluded from qPCR analysis if they were calculated to be outliers by the "identify outliers" function. No other data were excluded from analyses.
Replication	All data presented in this study are representative results of at least three independent experiments.
Randomization	Samples and animal subjects were allocated randomly.
Blinding	Data acquisition was performed in a blinded fashion.

Reporting for specific materials, systems and methods

We require information from authors about some types of materials, experimental systems and methods used in many studies. Here, indicate whether each material, system or method listed is relevant to your study. If you are not sure if a list item applies to your research, read the appropriate section before selecting a response.

Materials & experimental systems

n/a	Involved in the study	
☐	☒	Antibodies
☐	☒	Eukaryotic cell lines
☒	☐	Palaeontology
☐	☒	Animals and other organisms
☐	☒	Human research participants
☒	☐	Clinical data

Methods

n/a	Involved in the study	
☒	☐	ChIP-seq
☒	☐	Flow cytometry
☒	☐	MRI-based neuroimaging

Antibodies

Antibodies used	Information for antibodies is provided in the Supplementary Table 3.
Validation	Validation information for BMPER and NPC1 is provided in this manuscript (Fig. 1b for BMPER, Fig. 4c for NPC1). Other antibodies, including that for IRS1, IR, Smad1, 5, 8, AKT and beta-actin, have been validated in the manufacturer's websites.

Eukaryotic cell lines

Policy information about cell lines

Cell line source(s)	Information for cell line is provided in the Supplementary Table 3.
Authentication	The isolated mouse primary hepatocytes were subjected for real-time PCR assays (i.e. transthyretin, CD45, colagen Ial and Tie2). The high level of transthyretin but very low level of CD45, collagen Ial and Tie2 would suggest the highly enriched hepatocytes.
Mycoplasma contamination	All cell lines were tested as mycoplasma negative.
Commonly misidentified lines (See ICLAC register)	n/a.

Animals and other organisms

Policy information about studies involving animals; ARRIVE guidelines recommended for reporting animal research

Laboratory animals	We used BMPERflox;flox; CAG-CreER+/- (WT or iKO), BMPERflox;flox; Cdh5-CreER+/- (eWT or eKO), IRflox/flox; CAG-CreER+/- and db/db male mice at 2-5 months old for diabetes studies. C57BL/6J male mice at 6-8 weeks old were used for hepatocyte isolation and insulin signaling experiments. Their sources are listed in Supplementary Table 3.
Wild animals	n/a.
Field-collected samples	n/a.

Ethics oversight	All experimental procedures on mice were performed according to the National Institutes of Health Guide for the Care and Use of Laboratory Animals and approved by the Institutional Committee for the Use of Animals in Research at Baylor College of Medicine. Plasma samples were obtained from the study (Khan IM et al. J. Clin. Endocrinol. Metab. 2016) that was approved by the Institutional Review Board of Baylor College of Medicine.

Note that full information on the approval of the study protocol must also be provided in the manuscript.

Human research participants

Policy information about studies involving human research participants

Population characteristics	Subjects with metabolic syndrome (MS) were identified by the Adult Treatment Panel III criteria (27): at least 3 of the following were present: 1) high-density lipoprotein cholesterol (HDL-C) below 40 mg/dl for men and below 50 mg/dl for women; 2) glucose of at least 100 mg/dl; 3) triglycerides of at least 150 mg/dl; 4) blood pressure (BP) of at least 130/85 mm Hg; 5) waist circumference more than 102 cm in men and more than 88 cm in women. Individuals with BMI 18.5–27.4 kg/m2 and fasting triglycerides lower than 150 mg/dl were used as normal controls.
Recruitment	Male and female volunteers were recruited at the Center for Cardiovascular Disease Prevention at Baylor College of Medicine, Houston, Texas, or by advertisement. 11 Subjects with metabolic syndrome and 11 healthy individuals were recruited. Individuals were excluded if they were younger than 18 years of age, pregnant, or breastfeeding, or had acute illnesses, chronic liver or renal disease, cancer, bone fractures, diabetes, endocrinologic causes of obesity, obvious inflammation, or a history of myocardial infarction within the past 6 months or any hospitalization within the previous 2 months. Informed consent was obtained from all subjects.
Ethics oversight	The clinical study was approved by the Institutional Review Board for Baylor College of Medicine and Affiliated Hospitals. It is not a clinical trial and not prospectively registered.

Note that full information on the approval of the study protocol must also be provided in the manuscript.
